# Supplementary material for: T Cell Homeostasis Disturbances in a Cohort of Long-Term Elite Controllers of HIV Infection
Source: Int J Mol Sci. 2024 May 29;25(11):5937. doi: 10.3390/ijms25115937 (PMC11172696; doi:10.3390/ijms25115937)
Supplement: Supplementary file 1 [file ijms-25-05937-s001.zip › ijms-2991559-supplementary.pdf]

Supplementary Table S1. Phenotype of different functional traits of T cells analyzed in the study.

Functional trait of T cells	Markers combination (phenotype)	References
Maturation stage		43,44
Naive	CD45RA+CCR7+CD28+	
Central memory	CD45RA-CCR7+CD28+	
Effector memory	CD45RA-CCR7-CD28-	
TEMRA	CD45RA+CCR7-CD28-	
Recent thymic emigrants (RTEs)	CD45RA+CCR7+CD28+CD31+	45
Activation	CD38+HLADR+	46, 47
Exhaustion	PD1+ and/or Tim-3+ and/or CTLA4+ and/or TIGIT+	48
Senescence	CD28-CD57+	15, 49
Apoptosis	CD28-CD95+	50
Homeostatic potential	CD127+	51
T regulatory cells	CD127-CD25+CD39+/-	52, 53, 54
Th1/Tc1 cells	CXCR3+CCR6-	55, 56
Th2/Tc2 cells	CXCR3-CCR6-	56, 57
Th17/Tc17 cells	CXCR3-CCR6+	55, 56, 57
pTfh cells	CXCR5+	58

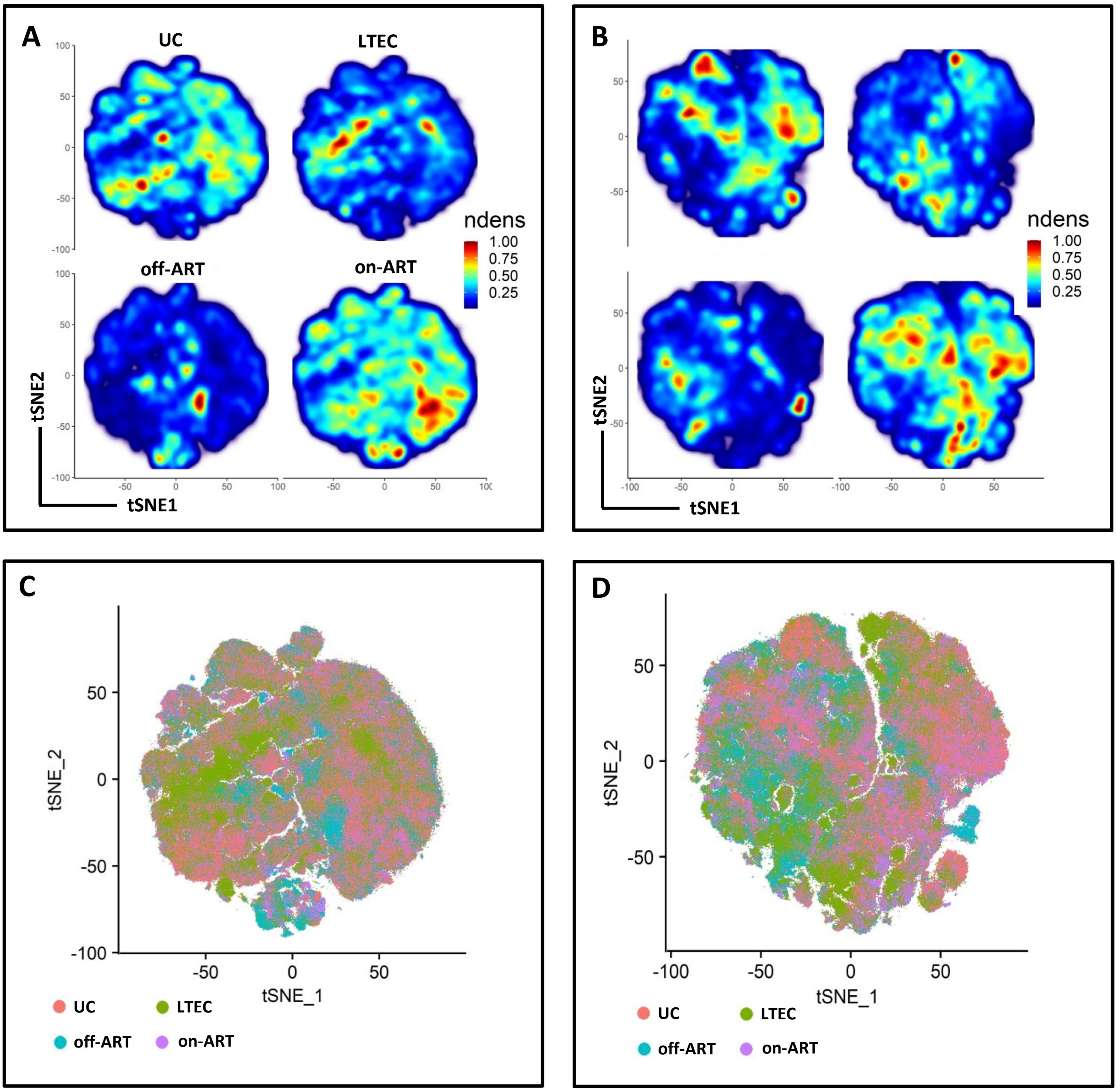

Supplementary figureS1. Upper graphs) tSNE maps colored by normalized density of events and split by study group. Maps colored by density help us to visualize where CD4 (**A**) or CD8 (**B**) events are more condensed in each study group and thus revealing inter-group differences in the distribution of events across the tSNE map. Normalized densities (ndens) were scaled to a maximum of 1 in each map, so that we can compare between different study groups independently of the number of samples analyzed (and thus the number of events) in each group. **Lower graphs)** tSNE map showing the distribution of CD4 (**C**) or CD8 (**D**) events across the map according to the study group (events from the different study groups are color-coded as shwon in the figure). An uneven distribution of the study groups across the map can be observed with events of certain study groups more abundant in certain areas of the map.

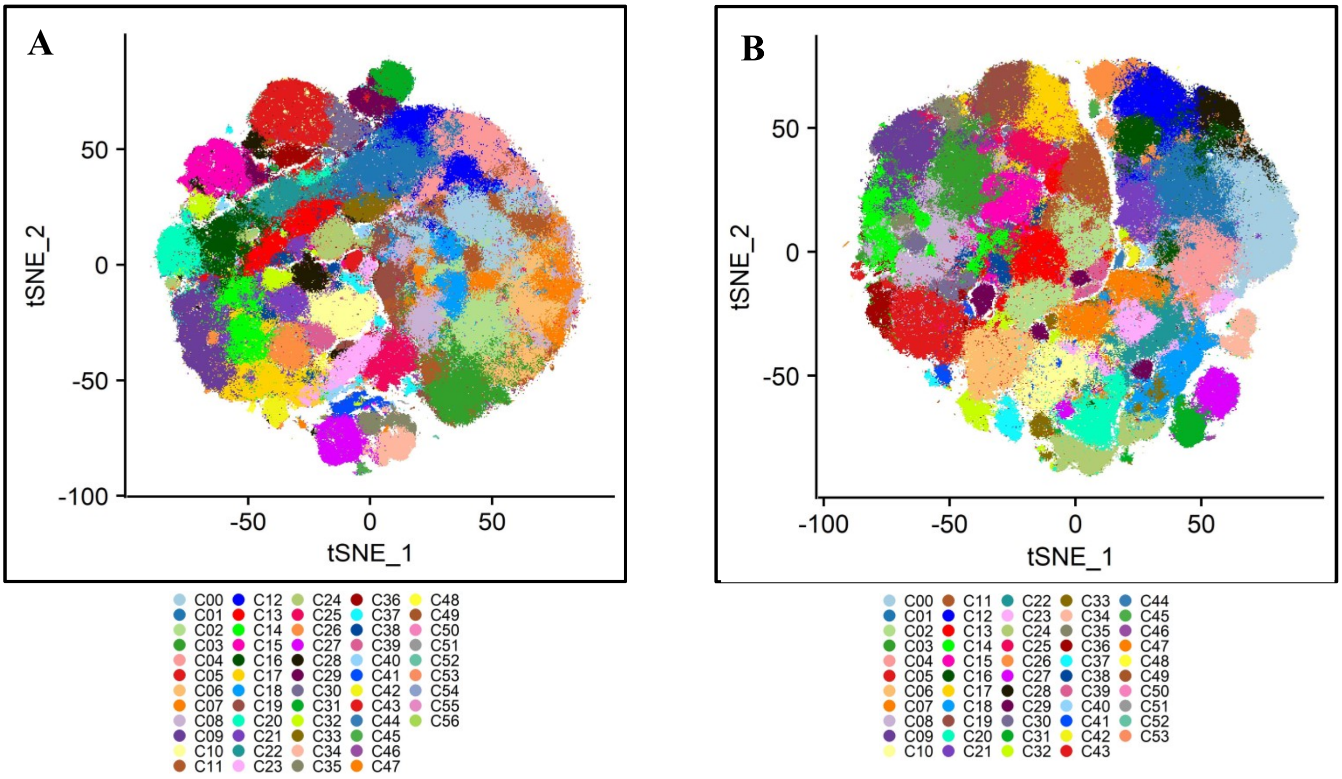

Supplementary figureS2. Louvain clustering of tSNE map, showing 57 different clusters of CD4 (**A**) and 54 different clusters of CD8 (**B**) T cells. The panels at the bottom indicate the color-coding of each cluster.

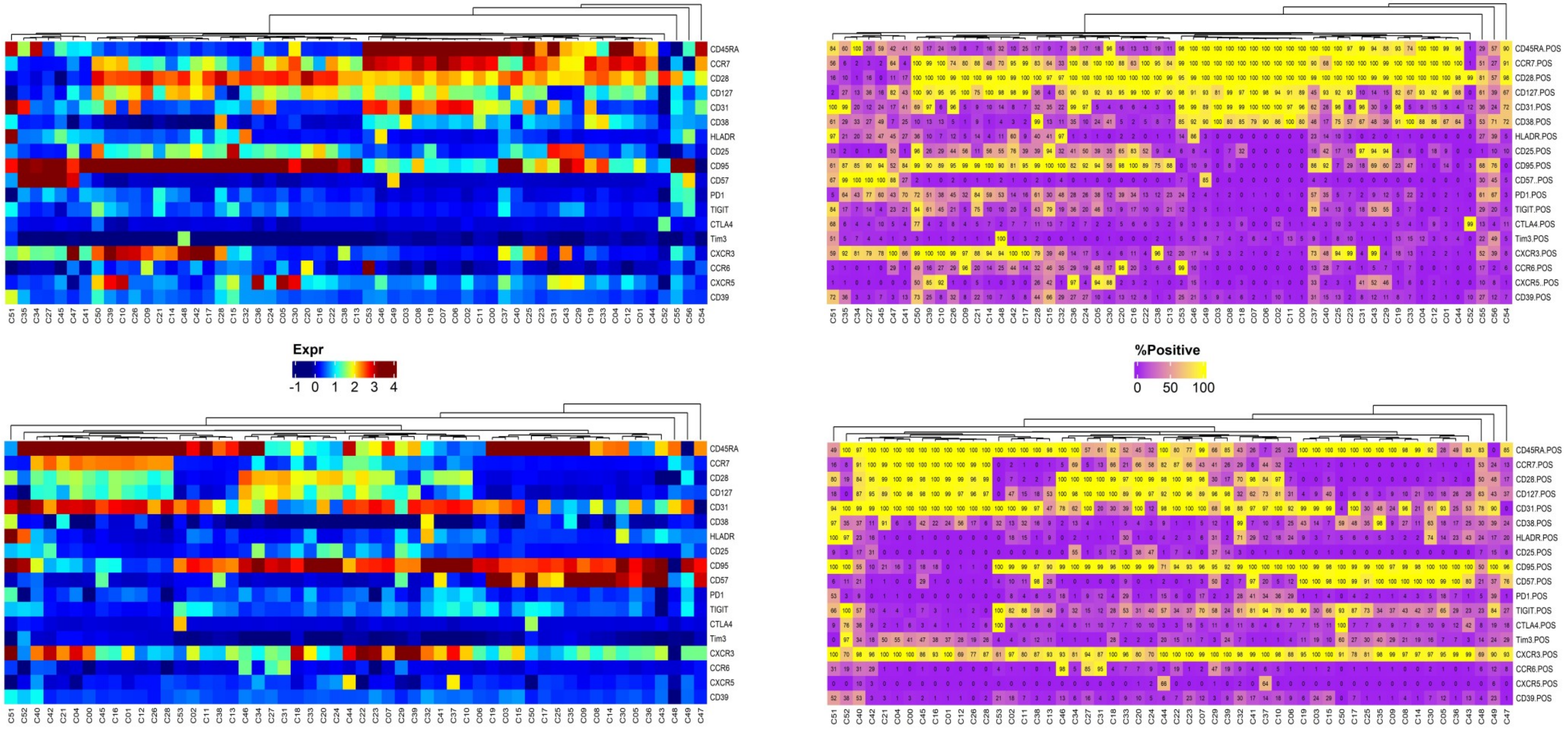

Supplementary figureS3. Left side) Heatmaps representing the expression level of different markers by each specific cluster of CD4 (up) and CD8 (down) T cells. Relative level of expression for each marker is color-coded from deep blue (lowest level of expression) to deep red (highest level of expression). The dendrogram at the top of the maps shows the grouping of clusters by similarity of markers expression. **Right side)** Heatmaps representing the percentage of positive events for a specific marker in each cluster of CD4 (up) and CD8 (down) T cells. Level of positivity (percentage of positive events) is color-coded from deep purple (lowest level of positivity) to yellow (highest level of positivity). Number inside each cell of the map indicates the percentage of positive events for a specific marker in a specific cluster. The dendrogram at the top of the map shows the grouping of clusters by similarity of percentage of positivity for each marker. In all maps, each column represents a specific cluster and each file a specific marker.

Supplementary table S2. Levels of CD4 T cells clusters showing significant differences (adjusted p-value<0.05) between study groups.

	Study group				
**Cluster**	**UC**	**LTEC**	**offART**	**onART**	**p<0.05**
C00	**4,28** [3,04 - 5,85]	**6,95** [4,65 - 8,87]	**1,02** [0,86 - 1,71]	**3,02** [1,71 - 5,30]	a, b, d, e
C02	**5,63** [3,52 - 6,58]	**2,62** [2,08 - 3,88]	**1,73** [1,40 - 2,82]	**5,72** [4,79 - 6,92]	a, b, e, f
C08	**1,32** [0,52 - 2,52]	**1,45** [0,85 - 2,04]	**5,024** [3,67 - 26,2]	**2,06** [1,03 - 3,83]	b, d
C09	**3** [2,27 - 4,50]	**3,62** [1,37 - 5,92]	**0,86** [0,57 - 1,06]	**1,7** [1,47 - 3,26]	b, d, f
C12	**3,62** [2,50 - 4,85]	**1,92** [0,81 - 2,79]	**1,67** [1,06 - 2,11]	**3,17** [2,12 - 3,79]	a, b
C13	**0,68** [0,52 - 1,0]	**4,12** [1,92 - 9,21]	**0,43** [0,28 - 0,48]	**0,85** [0,74 - 0,99]	a, b, d, e, f
C14	**2,4** [1,76 - 3,54]	**2,33** [1,46 - 4,35]	**0,8** [0,38 - 1,17]	**2,38** [1,59 - 2,94]	b, d, f
C17	**2,5** [2,05 - 3,82]	**2,39** [0,93 - 3,02]	**0,82** [0,54 - 1,32]	**2,88** [1,94 - 3,44]	b, d, f
C18	**2,76** [1,62 - 4,36]	**0,91** [0,65 - 1,20]	**1,24** [0,94 - 1,85]	**1,94** [1,30 - 3,66]	a, e
C21	**1,46** [1,05 - 1,88]	**2,82** [1,74 - 3,98]	**2,6** [1,62 - 3,28]	**1,59** [1,1 - 2,28]	a
C22	**2,56** [2 - 2,87]	**1,77** [0,93 - 2,85]	**0,85** [0,44 - 1,9]	**1,27** [0,96 - 2,12]	b
C24	**2,24** [1,75 - 3,41]	**1,07** [0,86 - 1,64]	**1,04** [0,54 - 1,48]	**1,8** [1,25 - 2,92]	a, b
C26	**2,94** [1,44 - 3,76]	**1,07** [0,48 - 1,73]	**0,89** [0,63 - 1,02]	**2** [1,45 - 3,02]	a, b, e, f
C28	**1,04** [0,78 - 1,31]	**1,3** [1,04 - 1,78]	**2,63** [2,22 - 2,81]	**1,26** [1,04 - 1,69]	b, d, f
C34	**0,08** [0,02 - 0,6]	**0,11** [0,04 - 0,4]	**1,52** [0,5 - 4]	**0,66** [0,2 - 2,31]	b, d
C35	**0,12** [0,04 - 0,86]	**0,13** [0,03 - 0,29]	**0,71** [0,48 - 2,61]	**1,14** [0,28 - 2,56]	e
C36	**1,35** [0,81 - 1,77]	**0,21** [0,13 - 0,39]	**0,58** [0,31 - 0,63]	**1,11** [0,69 - 1,33]	a, b, e, f
C37	**0,32** [0,26 - 0,54]	**0,23** [0,15 - 0,35]	**0,76** [0,75 - 1,96]	**0,48** [0,32 - 0,57]	b, d, e
C38	**0,18** [0,14 - 0,27]	**1,52** [0,77 - 2,62]	**0,11** [0,05 - 0,15]	**0,24** [0,2 - 0,29]	a, b, d, e, f
C39	**1,08** [0,57 - 1,42]	**0,42** [0,19 - 0,56]	**0,52** [0,28 - 0,63]	**0,65** [0,51 - 1,04]	a, b, e
C42	**0,3** [0,2 - 0,38]	**0,68** [0,38 - 1,70]	**0,15** [0,08 - 0,25]	**0,21** [0,1 - 0,32]	a, d, e
C43	**0,56** [0,43 - 0,75]	**0,34** [0,22 - 0,5]	**0,6** [0,3 - 0,7]	**0,62** [0,47 - 0,73]	a, e
C44	**0,05** [0,04 - 0,11]	**0,32** [0,19 - 0,72]	**0,01** [0 - 0,02]	**0,07** [0,03 - 0,11]	a, b, d, e, f
C45	**0** [0 - 0,01]	**0** [0 - 0,02]	**0,03** [0 - 0,68]	**0,01** [0,01 - 0,05]	c
C51	**0,01** [0 - 0,02]	**0,03** [0,02 - 0,05]	**0,02** [0,01 - 0,04]	**0,02** [0,01 - 0,04]	a
C52	**0,01** [0 - 0,02]	**0** [0 - 0,01]	**0,01** [0,01 - 0,02]	**0,03** [0,01 - 0,09]	e

Levels are given as **median** (Q1-Q3) and expressed as percentage over total CD4 T cells

a: LTEC vs. UC; **b:** offART vs. UC; **c:** onART vs. UC; **d:** LTEC vs. offART; **e:** LTEC vs. onART; f: offART vs. onART

Supplementary table S3. Levels of CD8 T cells clusters showing significant differences (adjusted p-value<0.05) between study groups.

	Study group				
**Cluster**	**UC**	**LTEC**	**offART**	**onART**	**p<0.05**
C00	**8,74**<br>[4,37 - 11,83]	**1,96**<br>[1,15 - 2,7]	**1,64**<br>[0,67 - 3,57]	**4,89**<br>[2,85 - 9,86]	a, b, e
C01	**7,12**<br>[5,3 - 10]	**2,72**<br>[1,62 - 5,67]	**0,96**<br>[0,48 - 1,78]	**6,13**<br>[1,98 - 7,98]	a, b, d, f
C04	**5,71**<br>[2,99 - 8,1]	**1,3**<br>[0,32 - 2,07]	**2,66**<br>[0,57 - 3,02]	**4,14**<br>[1,81 - 6,5]	a, e
C06	**0,56**<br>[0,25 - 1,23]	**3,32**<br>[1,81 - 7,67]	**4,06**<br>[1,48 - 9,89]	**1,09**<br>[0,54 - 1,89]	a, b, e
C11	**1,39**<br>[1,04 - 2,25]	**3,12**<br>[2,09 - 4,16]	**3,51**<br>[2,81 - 5,08]	**2,08**<br>[1,1 - 4,56]	a
C12	**3,15**<br>[2,31 - 4,45]	**3,85**<br>[1,83 - 5,99]	**1,35**<br>[0,58 - 1,8]	**2,36**<br>[1,46 - 4,36]	b, d
C15	**2,79**<br>[1,81 - 3,88]	**0,77**<br>[0,41 - 1,43]	**2,17**<br>[1,04 - 3,01]	**3,43**<br>[2,03 - 5,63]	a, e
C18	**1,7**<br>[1,06 - 3,81]	**1,74**<br>[1,35 - 3,81]	**1,03**<br>[0,96 - 1,32]	**2,47**<br>[2,02 - 4,2]	f
C20	**1,08**<br>[0,68 - 2,66]	**1,78**<br>[0,88 - 3,31]	**0,63**<br>[0,3 - 0,92]	**0,73**<br>[0,5 - 2,58]	d
C22	**3,09**<br>[2,26 - 4,21]	**1,34**<br>[0,88 - 2,1]	**1,15**<br>[0,22 - 1,84]	**2,6**<br>[1,86 - 4,47]	a, b, e, f
C23	**3,08**<br>[2,02 - 4,12]	**0,93**<br>[0,43 - 1,34]	**0,82**<br>[0,34 - 1,2]	**1,84**<br>[1,58 - 3,66]	a, b, e, f
C25	**0,59**<br>[0,17 - 1,51]	**1,37**<br>[0,89 - 1,73]	**3,61**<br>[1,56 - 4,39]	**2,12**<br>[0,51 - 2,84]	b
C26	**0,49**<br>[0,42 - 0,97]	**3,07**<br>[2,17 - 4,15]	**0,18**<br>[0,13 - 0,38]	**0,47**<br>[0,33 - 0,81]	a, b, d, e
C27	**2,14**<br>[1,15 - 3,13]	**0,73**<br>[0,45 - 2,12]	**0,35**<br>[0,18 - 0,71]	**0,55**<br>[0,36 - 1,27]	b
C28	**1,91**<br>[1,37 - 2,4]	**0,93**<br>[0,58 - 1,75]	**0,34**<br>[0,18 - 0,67]	**0,89**<br>[0,78 - 1,77]	b, d, f
C29	**0,62**<br>[0,4 - 0,96]	**1,97**<br>[0,92 - 4,78]	**0,98**<br>[0,61 - 1,19]	**0,69**<br>[0,3 - 0,83]	a, d, e
C30	**0,12**<br>[0,07 - 0,25]	**0,58**<br>[0,33 - 1,3]	**1,98**<br>[1,17 - 5,2]	**0,44**<br>[0,32 - 0,7]	a, b, c, d, f
C32	**0,4**<br>[0,29 - 0,52]	**1,03**<br>[0,79 - 1,26]	**2,32**<br>[1,84 - 3,77]	**0,46**<br>[0,36 - 0,68]	a, b, d, e, f
C35	**0,19**<br>[0,13 - 0,41]	**0,66**<br>[0,3 - 1,25]	**1,49**<br>[1,12 - 4,53]	**0,23**<br>0,13 - 0,75]	b, f
C38	**0,19**<br>[0,11 - 0,51]	**0,78**<br>[0,59 - 1,42]	**1,23**<br>[0,71 - 1,48]	**0,63**<br>[0,2 - 1]	a
C39	**0,87**<br>[0,54 - 1,08]	**0,36**<br>[0,23 - 0,81]	**0,16**<br>[0,05 - 0,22]	**0,87**<br>[0,55 - 1,08]	b, d, f
C45	**0,05**<br>[0,05 - 0,07]	**0,07**<br>[0,03 - 0,14]	**0,02**<br>[0 - 0,04]	**0,03**<br>[0,02 - 0,12]	b

Levels are given as **median** (Q1-Q3) and expressed as percentage over total CD4 T cells

a: LTEC vs. UC; **b:** offART vs. UC; **c:** onART vs. UC; **d:** LTEC vs. offART; **e:** LTEC vs. onART; **f**: offART vs. onART

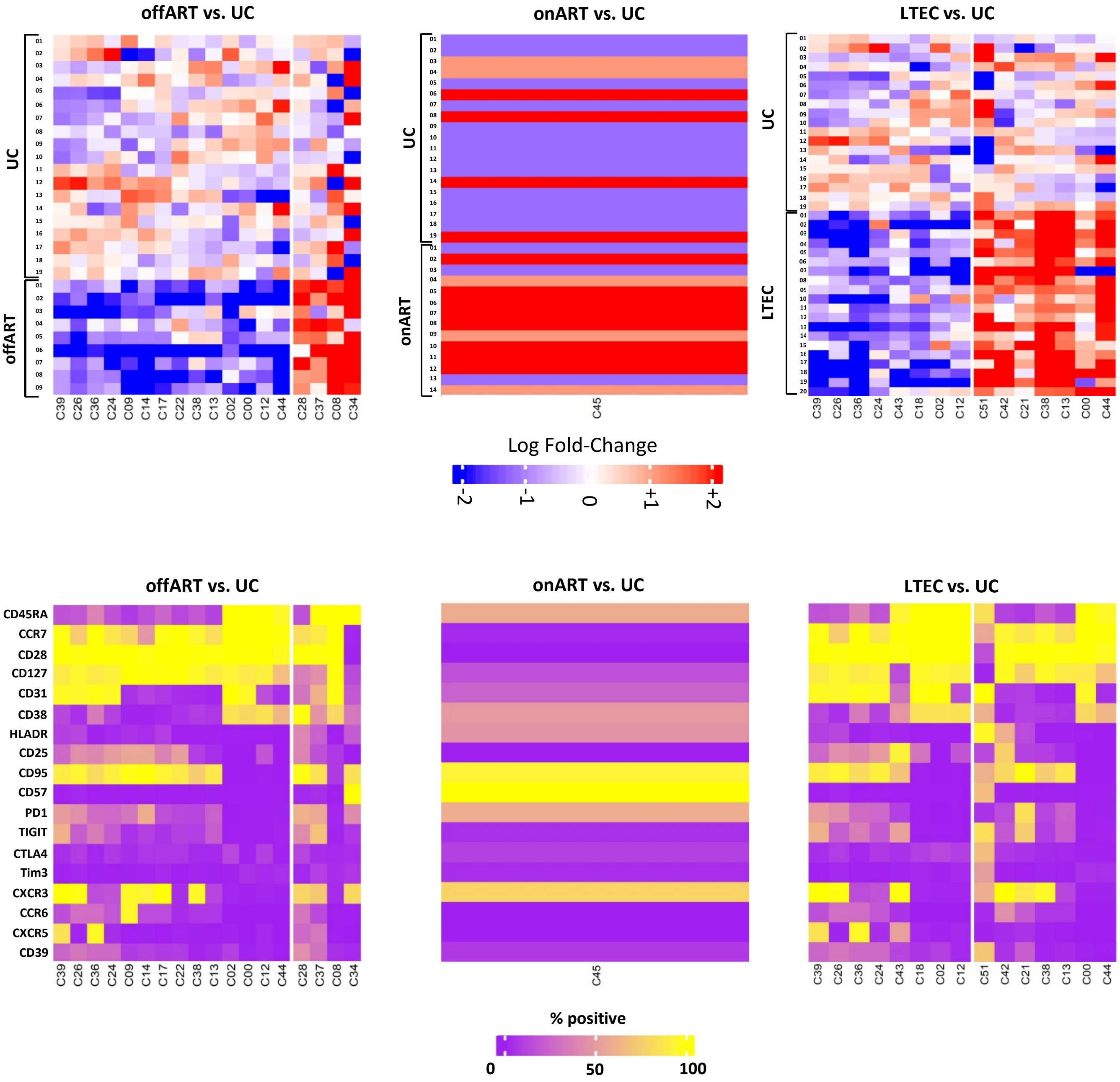

Supplementary figureS4. Top) Heatmaps of expression levels of the clusters of CD4 T cells that are differentially expressed between PLWH groups and UC group. The level of expression is expressed as LogFold-change taking the median value in the UC group as reference. Blue color indicates a diminished expression and red color an increased expression in each individual sample compared to the median value obtained in UC samples. In the heatmaps, each column represents a cluster and each row represents a sample. **Bottom)** Heatmaps representing the level of positivity (expressed as percentage of positive events) for a specific marker in each of the clusters of CD4 T cells differentially expressed in PLWH groups compared to UC group. Positivity for each marker was defined based on a manual gating (as explained in material and mehods). Level of positivity is color-coded from deep purple (lowest level of positivity) to yellow (highest level of positivity). In the heatmaps, each column represents a cluster and each row represents a marker.

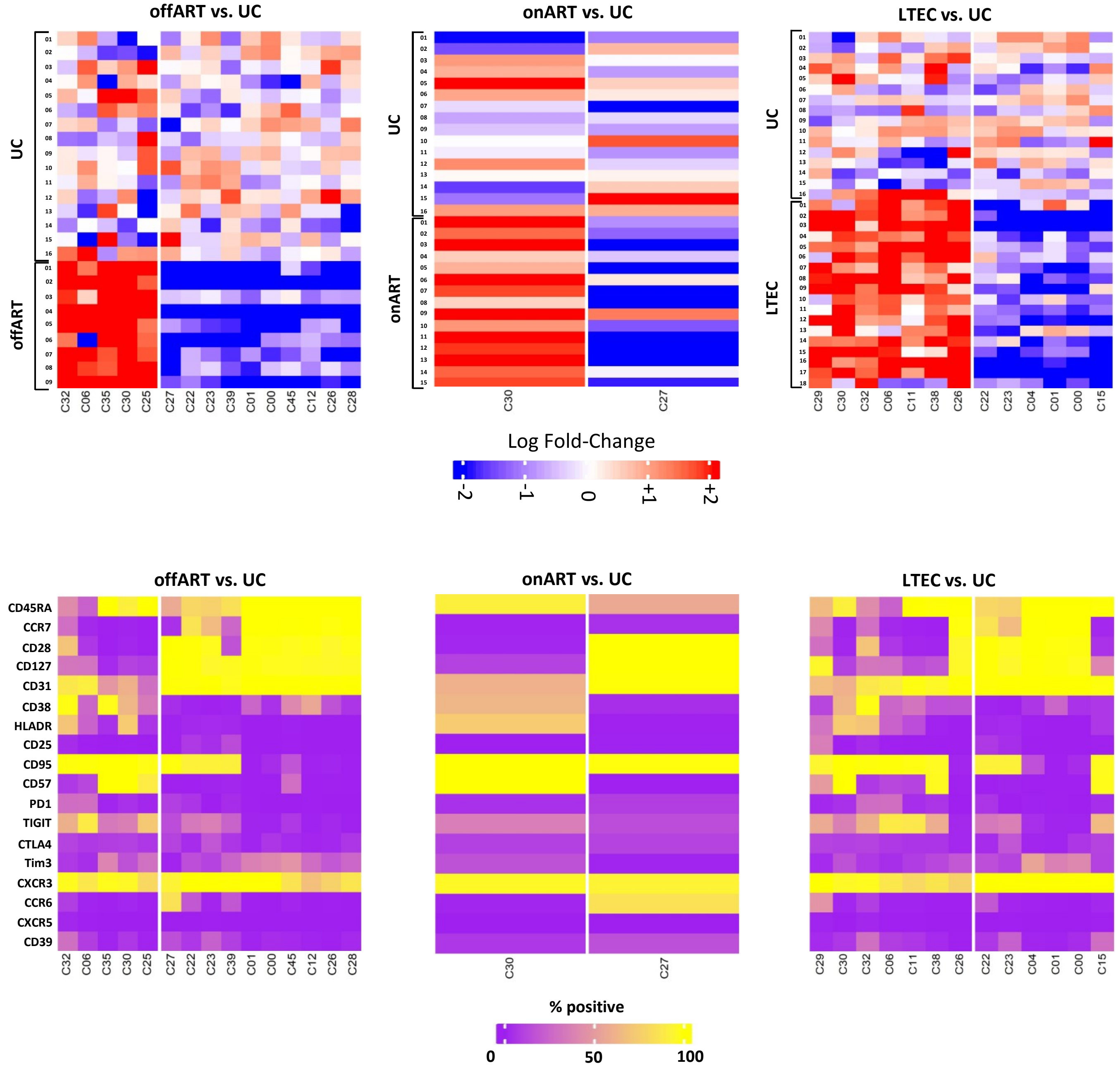

Supplementary figureS5. Top) Heatmaps of expression levels of the clusters of CD8 T cells that are differentially expressed between PLWH groups and UC group. The level of expression is expressed as LogFold-change taking the median value in the UC group as reference. Blue color indicates a diminished expression and red color an increased expression in each individual sample compared to the median value obtained in UC samples. In the heatmaps, each column represents a cluster and each row represents a sample. **Bottom)** Heatmaps representing the level of positivity (expressed as percentage of positive events) for a specific marker in each of the clusters of CD8 T cells differentially expressed in PLWH groups compared to UC group. Positivity for each marker was defined based on a manual gating (as explained in material and mehods). Level of positivity is color-coded from deep purple (lowest level of positivity) to yellow (highest level of positivity). In the heatmaps, each column represents a cluster and each row represents a marker.

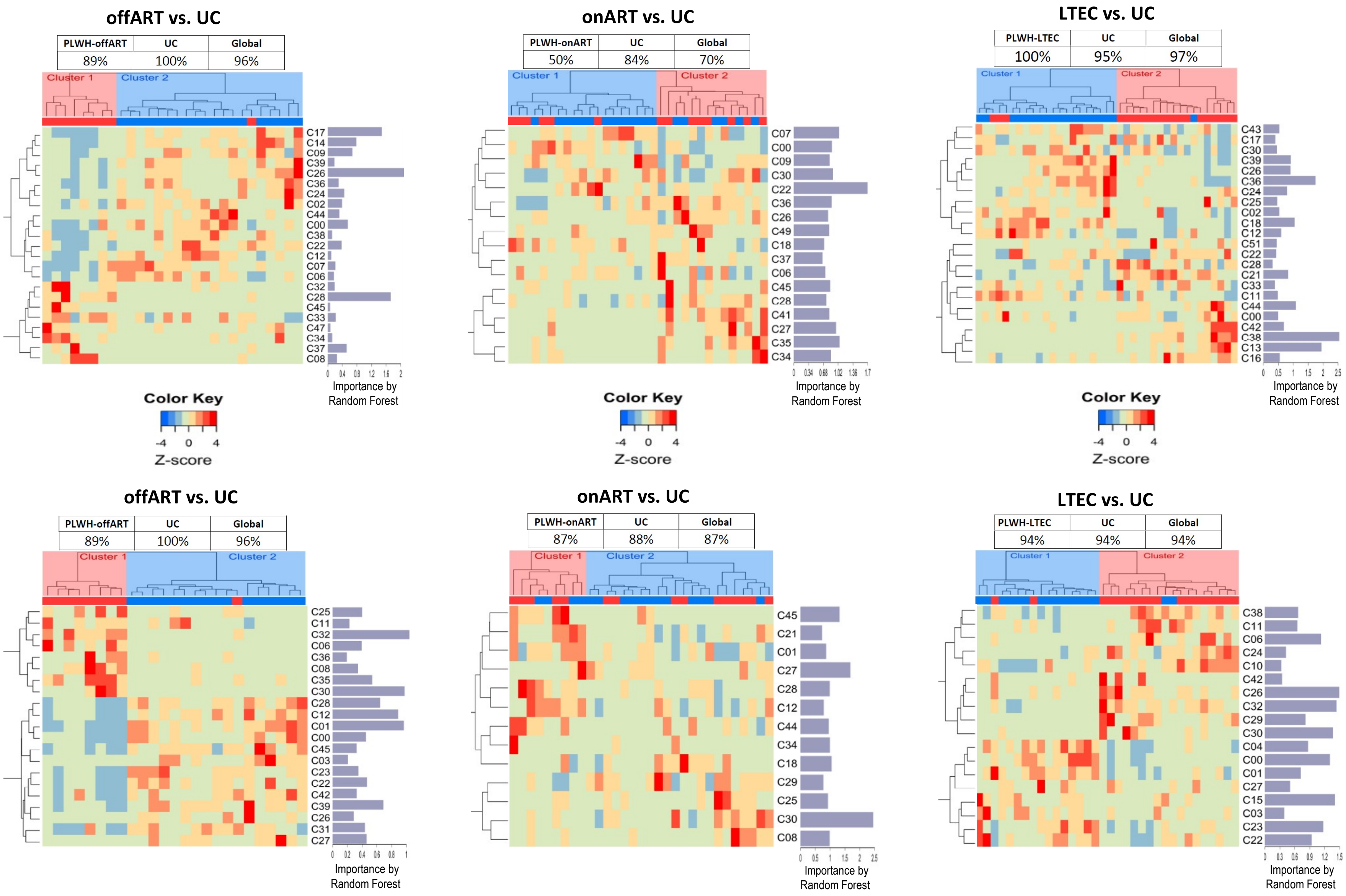

Supplementary figureS6. Heatmaps showing the expression level (Z-score) of those CD4 (**upper row**) and CD8 (**lower row**) T cells clusters that best discriminate between PLWH groups and UC group according to the random forest classification model. Tables on top of heatmap show the percentage of correct classification of the different study groups by the random forest model. In the heatmaps, each column represents a study participant and each row a cell cluster. Dendrograms on the top of the maps show the clustering of study participants with blue color representing UC participants and red color PLWH participants. Dendrograms on the left of the maps show the grouping of cell clusters. Bars on the right side of the maps show the importance of each cluster in the Random-Forest model of classification.

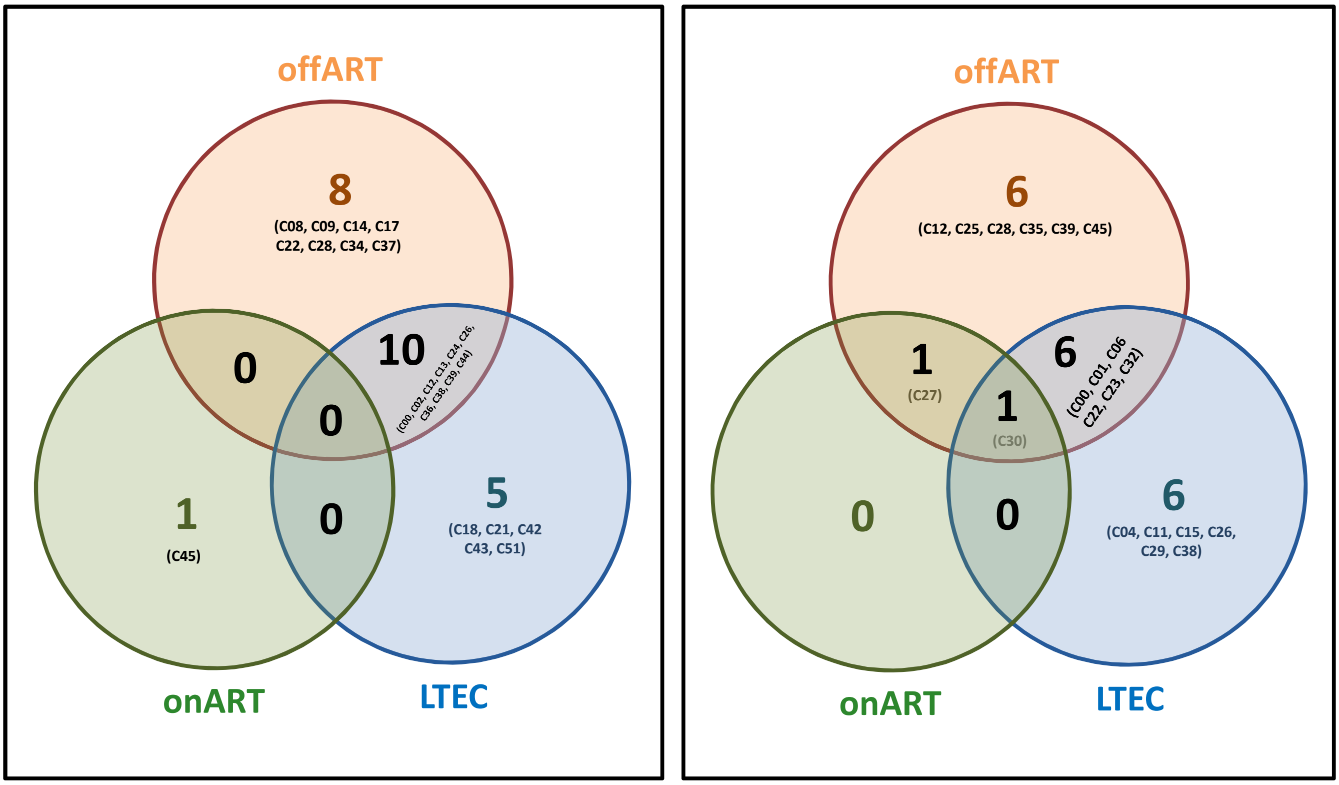

Supplementary figureS7. Venn diagram showing CD4 (**left**) and CD8 (**right**) T cells clusters differentially expressed in PLWH groups compared to UC group taken as reference. Each circle represents a PLWH group and numbers inside circles represent the number (and identification codes in parentheses) of clusters differentially expressed in each PLWH group with respect to UC group. Numbers inside overlapping areas between pairs of circles represent those clusters with differential expression that are shared between that pair of PLWH groups. Number inside the area overlapping the three circles represent those clusters with differential expression that are shared between the three PLWH groups. Numbers out of the overlapping areas represent clusters with differential expression only in that PLWH group.

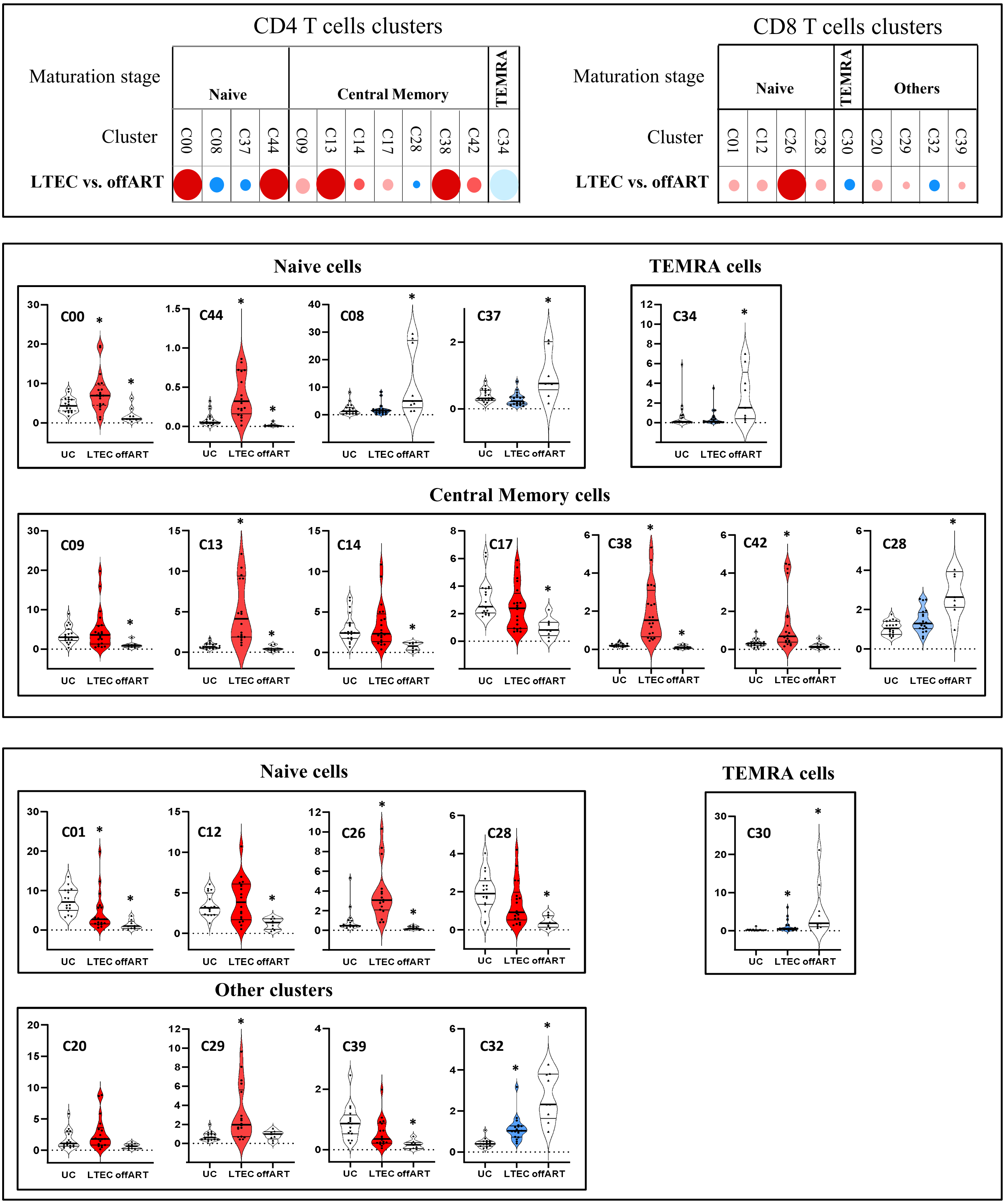

Supplementary figure S8. Upper graphs) Schematic representation (bubble diagram) of CD4 and CD8 T cells clusters with significant differences between LTEC and offART groups. Clusters are grouped according to the maturation stage. Each dot in the diagram represents a significant difference with respect to offART group. The size of the dot indicates the degree of difference in fold-change: <2, 2-3, 3-4, 4-5 and >5; from the smallest to the biggest size dot. Red colors indicate increase and blue colors indicate decrease with respect to offART group. Level of statistical significance (corrected p-value) is indicated by the color tone: 0.05-0.01, 0.01-0.001, and <0.001; for the light, medium and dark tone respectively. **Middle and lower graphs)** Violin-plots graphs of the levels of each cluster, expressed as percentage over total CD4 (middle graphs) or CD8 (lower graphs) T cells, in the UC, LTEC and offART groups. Clusters are grouped according to maturation stage. In each violin-plot graph, blue-colored violins show a statistically significant decrease and red-colored violins a statistically significant increase with respect to offART group. The asterisk symbol (*) over the violin plots indicates a statistically significant difference with respect to UC reference group.

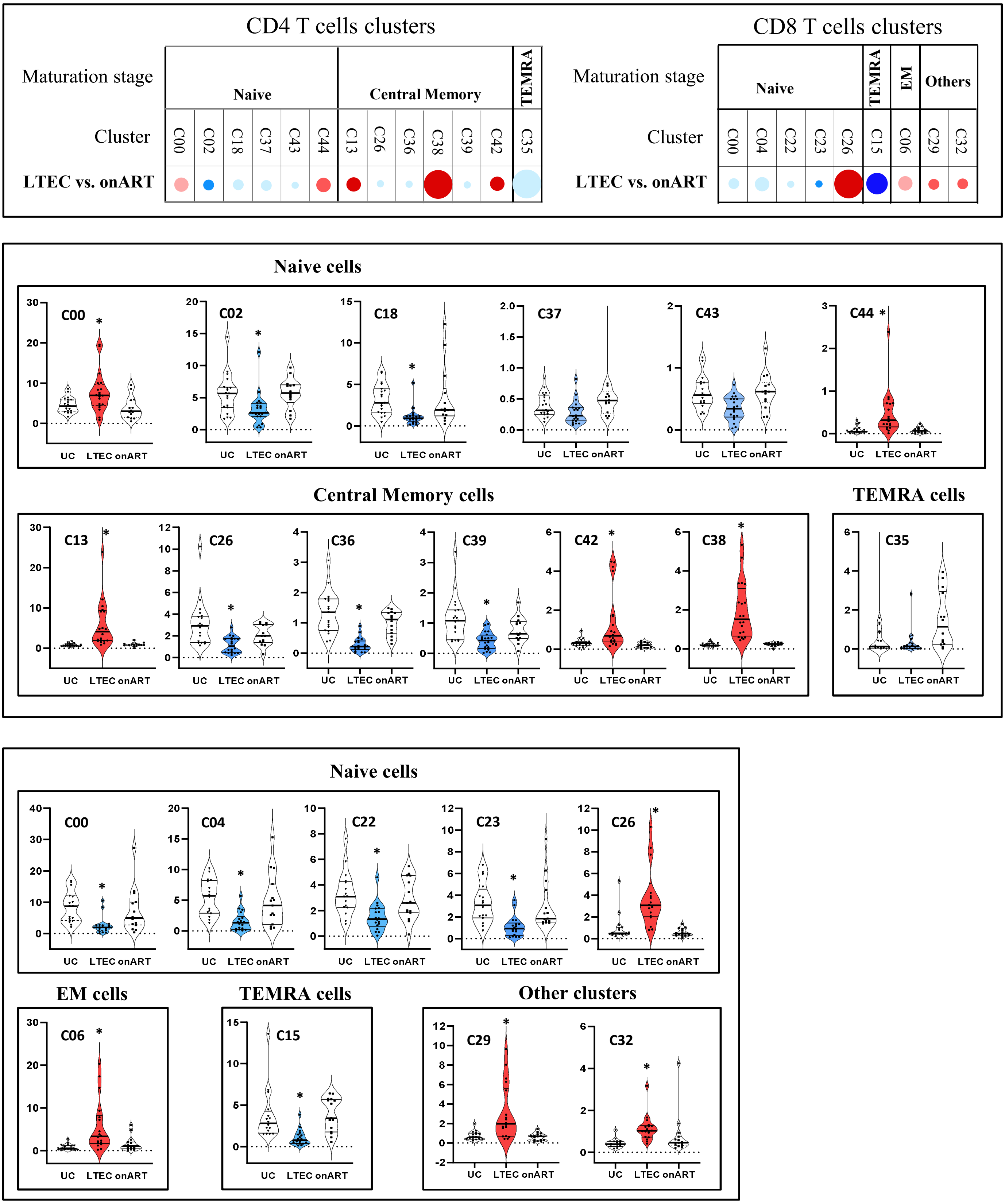

Supplementary figure S9. Upper graphs) Schematic representation (bubble diagram) of CD4 and CD8 T cells clusters with significant differences between LTEC and onART groups. Clusters are grouped according to the maturation stage. Each dot in the diagram represents a significant difference with respect to onART group. The size of the dot indicates the degree of difference in fold-change: <2, 2-3, 3-4, 4-5 and >5; from the smallest to the biggest size dot. Red colors indicate increase and blue colors indicate decrease with respect to onART group. Level of statistical significance (corrected p-value) is indicated by the color tone: 0.05-0.01, 0.01-0.001, and <0.001; for the light, medium and dark tone respectively. **Middle and lower graphs)** Violin-plots graphs of the levels of each cluster, expressed as percentage over total CD4 (middle graphs) or CD8 (lower graphs) T cells, in the UC, LTEC and onART groups. Clusters are grouped according to maturation stage. In each violin-plot graph, blue-colored violins show a statistically significant decrease and red-colored violins a statistically significant increase with respect to onART group. The asterisk symbol (*) over the violin plots indicates a statistically significant difference with respect to UC reference group.

Supplementary Table S4. Monoclonal antibodies and fluorochromes used in the study.

Antibody	Fluorochrome	Clone	Provider
CD3	BV570	UCHT1	Biolegend
CD4	PercPCy5.5	RPA-T4	Biolegend
CD8	BUV805	SK1	BD Biosciences
CD45RA	BUV395	5H9	BD Biosciences
CD28	BV650	CD28.2	Biolegend
CCR7	BV421	G043H7	Biolegend
CD31	BUV737	WM59	BD Biosciences
CD38	APCFIRE810	HIT2	Biolegend
HLA-DR	APCFIRE750	L243	Biolegend
PD-1	BV785	EH122H7	Biolegend
Tim3	AF647	7D3	Biolegend
CTLA4	PEDAZZLE	BNI3	Biolegend
TIGIT	BV480	741182	BD Biosciences
CD95	PECy5	DX2	Biolegend
CXCR3	PECy7	G025H7	Biolegend
CCR6	BV711	G034E3	Biolegend
CD127	APCR700	HIL-7R-M21	BD Biosciences
CD25	PEAF700	CD25-3G10	Thermofisher
CD39	BUV661	TU66	BD Biosciences
CXCR5	PE	J252D4	Biolegend
CD57	FITC	HNK-1	Biolegend
-	Live/Dead Fixable blue		Thermofisher

Supplementary Methods

Clinical Centers and research groups which contribute to ECRIS cohort

Clinical centers:

Hospital Universitario de Valme (Sevilla): Juan Antonio Pineda, Eva Recio Sánchez, Fernando Lozano de León, Juan Macías, José Carlos Palomares, Manuel Parra, Jesús Gómez-Mateos.

Hospital General Universitario Santa Lucía (Cartagena): Onofre Juan Martínez-Madrid, Francisco Vera, Lorena Martínez.

Hospital Clinic de Barcelona (Barcelona): José M. Miró, José Luis Blanco, Felipe García, Esteban Martínez, Josep Mallolas, Montserrat Laguno, María Martínez, Berta Torres, Lorna Leal, Ainoa Ugarte, Flor Etcheverry, Irene Fernández.

Hospital General Universitario de Alicante (Alicante): Joaquín Portilla, Esperanza Merino, Sergio Reus, Vicente Boix, Livia Giner, Carmen Gadea, Irene Portilla, Maria Pampliega, Marcos Díez, Juan Carlos Rodríguez, Jose Sánchez-Payá.

Hospital Universitari de Bellvitge (Hospitalet de Llobregat): Daniel Podzamczer, Elena Ferrerm Arkaitz Imaz, Evan Van Den Eyncle, Silvana Di Yacovo, Maria Sumoy.

Hospital Universitario de Canarias (Santa Cruz de Tenerife): Juan Luis Gómez, Patricia Rodríguez, María Remedios Alemán, María del Mar Alonso, María Inmaculada Hernández, Felicitas Díaz-Flores, Dácil García, Ricardo Pelazas.

Hospital Carlos III (Madrid): Vicente Soriano, Pablo Labarga, Pablo Barreiro, Pablo Rivas, Francisco Blanco, Luz Martín Carbonero, Eugenia Vispo, Carmen Solera.

Hospital Universitario Central de Asturias (Oviedo): Victor Asensi, Eulalia Valle, José Antonio Cartón.

Hospital Doce de Octubre (Madrid): Rafael Rubio, Federico Pulido, Mariano Matarranz, Maria Lagarde, Guillermo Maestro, Rafael Rubio-Martín.

Hospital Universitario Donostia (San Sebastián): José Antonio Iribarren, Julio Arrizabalaga, María José Aramburu, Xabier Camino, Francisco Rodríguez-Arrondo, Miguel Ángel von Wichmann, Lidia Pascual Tomé, Miguel Ángel Goenaga, Mª Jesús Bustinduy, Harkaitz Azkune Galparsoro. Maialen Ibarguren, Mirian Aguado.

Hospital General Universitario de Elche (Elche): Félix Gutiérrez, Mar Masiá, Cristina López, Sergio Padilla, Andrés Navarro, Fernando Montolio, Catalina Robledano, Joan Gregori Colomé, Araceli Adsuar, Rafael Pascual, Federico Carlos, Maravillas Martinez.

Hospital Germans Trías i Pujol (Badalona): Roberto Muga, Jordi Tor, Arantza Sanvisens.

Hospital General Universitario Gregorio Marañón (Madrid): Juan Berenguer, Juan Carlos López Bernaldo de Quirós, Pilar Miralles, Isabel Gutiérrez, Margarita Ramírez, Belén Padilla, Paloma Gijón, Ana Carrero, Teresa Aldamiz-Echevarría, Francisco Tejerina, Francisco Jose Parras, Pascual Balsalobre, Cristina Diez.

Hospital Universitari de Tarragona Joan XXIII, IISPV, Universitat Rovira i Virgili (Tarragona): Francesc Vidal, Joaquín Peraire, Consuelo Viladés, Sergio Veloso, Montserrat

Vargas, Miguel López-Dupla, Montserrat Olona, Alba Aguilar, Joan Josep Sirvent, Verónica Alba, Olga Calavia.

Hospital Universitario La Fe (Valencia): Marta Montero, José Lacruz, Marino Blanes, Eva Calabuig, Sandra Cuellar, José López, Miguel Salavert.

Hospital Universitario La Paz/IdiPaz (Madrid): Juan González, Ignacio Bernardino de la Serna, José Ramón Arribas, María Luisa Montes, Jose Mª Peña, Blanca Arribas, Juan Miguel Castro, Fco Javier Zamora, Ignacio Pérez, Miriam Estébanez, Silvia García, Marta Díaz, Natalia Stella Alcáriz, Jesús Mingorance, Dolores Montero, Alicia González, Maria Isabel de José.

Hospital de la Princesa (Madrid): Ignacio de los Santos, Jesús Sanz, Ana Salas, Cristina Sarriá, Ana Gómez.

Hospital San Pedro-CIBIR (Logroño): José Antonio Oteo, José Ramón Blanco, Valvanera Ibarra, Luis Metola, Mercedes Sanz, Laura Pérez-Martínez.

Complejo Hospitalario de Navarra (Pamplona): María Rivero, Marina Itziar Casado, Jorge Alberto Díaz, Javier Uriz, Jesús Repáraz, Carmen Irigoyen, María Jesús Arraiza.

Hospital Parc Taulí (Sabadell): Ferrán Segura, María José Amengual, Gemma Navarro, Montserrat Sala, Manuel Cervantes, Valentín Pineda, Victor Segura, Marta Navarro, Esperanza Antón, Mª Merce Nogueras.

Hospital Ramón y Cajal (Madrid): Santiago Moreno, José Luis Casado, Fernando Dronda, Ana Moreno, María Jesús Pérez Elías, Dolores López, Carolina Gutiérrez, Beatriz Hernández, Nadia Madrid, Angel Lamas, Paloma Martí, Alberto de Diaz, Sergio Serrano, Lucas Donat.

Hospital Reina Sofía (Murcia): Alfredo Cano, Enrique Bernal, Ángeles Muñoz.

Hospital San Cecilio (Granada): Federico García, José Hernández, Alejandro Peña, Leopoldo Muñoz, Jorge Parra, Marta Alvarez, Natalia Chueca, Vicente Guillot, David Vinuesa, Jose Angel Fernández.

Centro Sanitario Sandoval (Madrid): Jorge Del Romero, Carmen Rodríguez, Teresa Puerta, Juan Carlos Carrió, Cristina González, Mar Vera, Juan Ballesteros.

Hospital Son Espases (Palma de Mallorca): Melchor Riera, María Peñaranda, María Leyes, Mº Angels Ribas, Antoni A Campins, Carmen Vidal, Leire Gil, Francisco Fanjul, Carmen Matinescu.

Hospital Universitario Virgen del Rocío (Sevilla): Manuel Leal, Pompeyo Viciana, Luis Fernando López-Cortés, Nuria Espinosa.

Research groups:

IIS-Fundación Jimenez Díaz, UAM: José Miguel Benito, Norma Rallón, Clara Restrepo, Alfonso Cabello, Miguel Gorgolas.

Infección viral e Inmunidad. ISCIII: Salvador Resino, Veronica Briz, Maria Angeles Jimenez, Maria Sonia Vazquez, Amanda Fernandez, Pilar García.

Hospital General Universitario Gregorio Marañón: Maria Angeles Muñoz, Javier Sanchez Rodriguez, Jose Luis Jimenez, Daniel Sepúlveda, Isabel García Merino, Irene Consuegra.

Hospital Clinic-IDIBAPS: Agathe León, Sonsoles Sánchez, Mireia Arnedo, Montserrat Plana, Nuria Climent, Felipe García.

Hospital Joan XXIII: Paco Vidal, Esther Rodriguez-Gallego, Consuelo Viladés, Joaquin Peraire

Centro Sandoval: Jorge Del Romero, Carmen Rodríguez, Mar Vera.

Fundación IrsiCaixa: José Esté, Esther Ballana, Miguel Angel Martinez, S Franco,María Nevot.

Hospital Ramón y Cajal: Alejandro Vallejo, Beatriz Sara Sastre, Santiago Moreno.

Virologia Molecular ISCIII: Maria Pernas, Concepción Casado, Cecilio López Galíndez

Inmunopatología del SIDA, ISCIII: Laura Capa, Mayte Perez-Olmeda, Pepe Alcami

Mutación y evolución de virus. Univ. Valencia: Rafael Sanjuán, José Manuel Cuevas

Hospital Virgen del Rocío: Ezequiel Ruiz-Mateos, Beatriz Dominguez-Molina, Laura Tarancón-Diez, Mohamed Rafii-El-Idrissi Benhnia, Maria José Polaino, Miguel Genebat, Pompeyo Viciana, Manuel Leal.

Hospital 12 de Octubre: Rafael Delgado, Olalla Sierra

Universidad de la Laguna: Agustín Valenzuela-Fernández.

Staining protocol for flow cytometry

One million of PBMCs were washed with 2 mL of phosphate-buffered saline (PBS) and stained for surface markers by sequential incubation, as follows: firstly cells were incubated with Live/dead fixable blue viability dye for 15 minutes in the dark at room temperature (RT). Secondly, cells were washed with 2ml of PBS and incubated with anti-CCR7 antibody for 10 minutes at RT in the dark. Thirdly, anti-CCR6, anti-CXCR3 and anti-CXCR5 antibodies were added to cell suspension and incubated for another 10 minutes RT in the dark. Lastly the rest of antibodies were added to cell suspension and incubated for 30 minutes at RT in the dark. Antibodies conjugated with Brilliant Violet were mixed with brilliant stain buffer before being added to the cell samples. Cells were washed with 2 mL of PBS and resuspended in 200 µL of PBS plus 0.5% of fetal bovine serum (FBS) for acquisition in an Aurora spectral flow cytometer (Cytek Biosciences, USA).

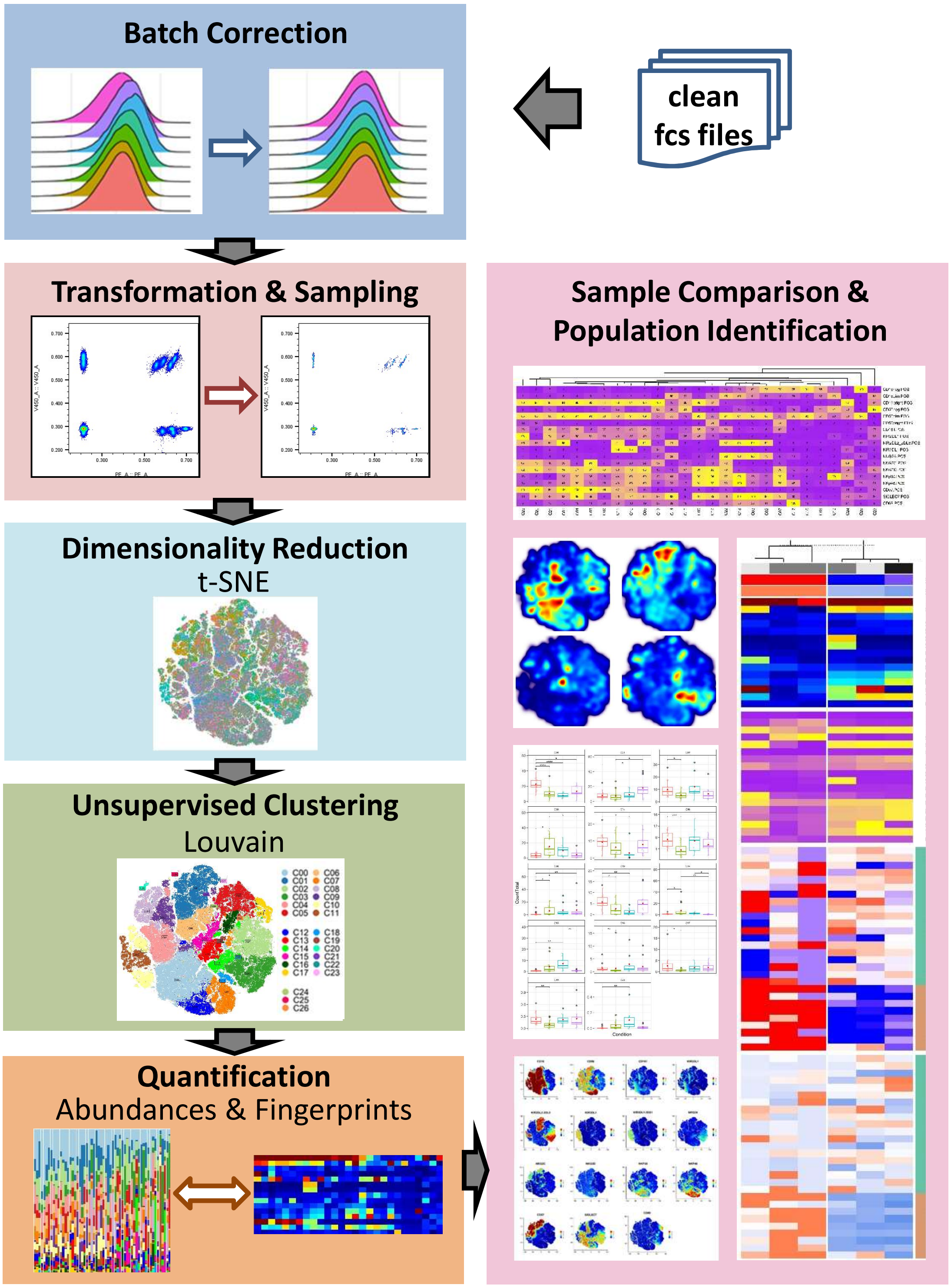

Supplementary figure S10. Pipeline for multidimensional analysis of flow cytometry data.
